# Characteristics of Posterior Cerebral Circulation in Patients With Intractable Hiccups: A Case Series

**DOI:** 10.1002/brb3.70978

**Published:** 2025-10-15

**Authors:** Tsukasa Kondo, Teruhiko Terasawa, Hideyuki Nakama

**Affiliations:** ^1^ Department of Emergency Medicine Yuai Memorial Hospital Koga Ibaraki Japan; ^2^ Department of Emergency and General Internal Medicine Fujita Health University School of Medicine Toyoake Aichi Japan; ^3^ Department of Neurosurgery Yuai Memorial Hospital Koga Ibaraki Japan

**Keywords:** Circle of Willis, intractable hiccups, posterior communicating artery, reflex

## Abstract

**Introduction:**

Certain vascular variations, including complete fetal‐type posterior cerebral artery variants, may underlie the mechanisms contributing to intractable hiccups.

**Methods:**

Brain magnetic resonance angiography was performed in 230 patients with intractable hiccups who visited our hiccup consultation clinic. Based on the proposed morphological classification system, vascular distribution patterns and frequencies of the Circle of Willis (CW) were compared with those in five previously reported healthy cohorts.

**Results:**

Of the 230 patients, 215 (93.5%) had an incomplete CW type, which was significantly higher than that observed in the healthy cohorts (45.0–74.6%; all *p* < 0.001). Hypoplasia or absence of both posterior communicating arteries, resulting in isolation of the anterior and posterior CW, was the most common phenotype (140/230, 60.9%). This frequency was also higher than that reported in each of the healthy cohorts (8.7–42.8%; all *p* < 0.001).

**Conclusions:**

Incomplete CW patterns were prevalent among individuals with intractable hiccups, particularly those isolating the anterior and posterior CW. These findings suggest a possible association between intractable hiccups, cerebrovascular variants, and changes in cerebral blood flow.

## Graphical abstract

This study investigated the potential association between intractable hiccups and cerebrovascular variants. Clinical and imaging data from 230 patients with intractable hiccups were analyzed. The findings revealed that posterior communicating artery (P‐com) deficiency and type e variations of the Circle of Willis (CW) were highly prevalent among patients.

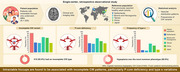

## 1 Introduction

Hiccups lasting longer than 1 month are referred to as intractable hiccups (Kondo et al. 2003). Cerebrovascular events have been reported alongside various etiologies associated with hiccups (Launois et al. 1993). Alterations in posterior cerebral circulation, including complete fetal posterior cerebral artery (PCA) variants, are associated with cerebral ischemia due to a reduction in blood supply to the PCA region (Lochner et al. 2011). Controversy exists regarding whether variant forms of the Circle of Willis (CW), particularly pathological one, contribute to reduced cerebral blood flow and ischemia. Such vascular variations may help explain the mechanisms underlying intractable hiccups. However, cerebrovascular variations (i.e., pattern and frequency) in this patient population have yet to be fully characterized. Although it is known that the hiccup center exists in the medulla oblongata, the location of the higher center that controls the hiccup center is unknown. This study may help identify the potential higher brain centers involved in the regulation of the hiccup reflex.

## 2 Methods

This retrospective observational study included patients with intractable hiccups who were treated during a hiccup consultation at the Yuai Memorial Hospital, a secondary community hospital in Japan. In 2017, a specialized outpatient clinic for intractable hiccups (hiccups lasting > 1 month) was established, where the first author (TK) was responsible for treating all the patients. All consecutive patients who visited the hiccup clinic between April 1, 2017 and March 31, 2021, and underwent magnetic resonance angiography (MRA) were included in this study. Patients who did not undergo MRA were excluded.

Clinical and imaging data were extracted through retrospective chart review. This study was approved by the Ethics Committee of the Yuai Memorial Hospital and conformed to the guidelines set in the Declaration of Helsinki. Informed consent was obtained from all participants.

**FIGURE 1:**
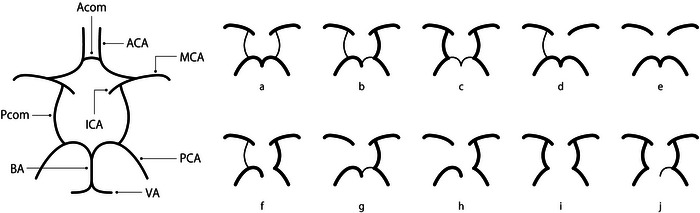
Anatomical structure of the arteries (large panel) and variation types (small panels, a–j) of the posterior portions of the Circle of Willis. Type‐e was the most common type in the case group. The anterior portions of the Circle of Willis showed very little variation and were therefore removed from the figure. Reproduced with permission to use copyrighted material from Reference 4. Variation types a–c have no defects in the Circle of Willis and are called complete types, whereas variation types d–j exhibit missing in the Circle of Willis and are called incomplete types. ACA: anterior cerebral artery; A‐com: anterior communicating artery; BA: basilar artery; MCA: middle cerebral artery; PCA: posterior cerebral artery; P‐com: posterior communicating artery; VA: vertebral artery.

Unless otherwise contraindicated, a 1.5 Tesla magnetic resonance imaging (MRI) scanner (GE Optima MR450w 1.5T; GE Healthcare, Chicago, IL, USA) was used to obtain unenhanced brain MRI scans to check for lesions, including those in the medulla oblongata. Using three‐dimensional time‐of‐flight MRA images, we identified the vascular distribution pattern of the CW and any cerebrovascular abnormalities, including stenosis and aneurysm formation. Subsequently, these anatomical variants were categorized into 10 subtypes (a–j) based on previously published parameters (Figure 1) (Krabbe‐Hartkamp et al. 1998).

The etiology of intractable hiccups can be multifocal and includes pathological conditions and lifestyle habits. Although we aimed to determine the most plausible underlying etiology given all available information, we accepted the existence of multiple underlying conditions that could induce and maintain intractable hiccupping. The underlying diseases were determined based on the patient's symptoms. For example, vagus or phrenic nerve irritation was defined as hiccups accompanied by coughing, nausea, or vomiting. Gastric etiologies were defined as symptoms such as upper abdominal pain or belching. Esophageal etiologies were defined as symptoms scored 8 or higher on the FSSG (Frequency Scale for the Symptoms of Gastroesophageal reflux disease) scale (Kusano M. et al. 2012). A whole‐body computed tomography scan was also performed from the neck to the pelvic organs in an effort to identify the causes of intractable hiccups.

Drug‐induced etiologies were defined as symptoms that occurred after the start of administration of a certain drug. In the case of medullary tumors, there are often no symptoms other than hiccups, so if a tumor was found near the medulla oblongata on an MRI image, it was considered to be the underlying disease.

Even if a disease was considered to be related to hiccups at the initial consultation, it was removed from the list of related diseases when it was considered to be less related at the later consultations. If the opposite was true, it was added to the list later.

All variables were descriptively analyzed as numbers and percentages, means and standard deviations, or medians and interquartile ranges, depending on their distribution.

To establish a reference population (i.e., controls), we adopted a stepwise approach and used data from five previously reported healthy adult cohorts, one from Japan (Ikeda et al. 2003) and others from the Netherlands (Krabbe‐Hartkamp et al. 1998), Bosnia and Herzegovina (Voljevica et al. 2005), China (Li et al. 2011), and Taiwan (Chen et al. 2004). First, to statistically screen for overall between‐group differences, we performed chi‐square tests to assess the heterogeneity of proportions across groups. Subsequently, to explore the reasons for the observed heterogeneity, we assessed the statistical differences in the frequency of a specific group of variant subtypes compared to that of all other groups combined. The focus groups included in the analysis were as follows: (i) Complete (types a–c) versus incomplete (types d–j) CW types; (ii) deficient types of posterior communicating arteries (P‐com; referred to as P‐com deficiency; types d, e, g, and h) versus non‐P‐com deficiency (types a–c, f, i, and j); and (iii) fetal types (types b, c, f–j) versus non‐fetal types (types a, d and e). The fetal type was defined as the type of circulation in which the P‐com branched from the internal carotid artery. Finally, we assessed the difference in the frequency of the most common variant group in the intractable hiccup cohort compared with that of the corresponding variant group in each control population. Subgroup analyses were performed based on sex and the presence or absence of cerebrovascular incidents. For missing data, we performed only a complete case analysis. All analyses were conducted using Stata 17.0 (Stata Corp., College Station, TX, USA), and a *p*‐value of < 0.05 was considered significant.

## 3 Results

Of the 249 patients who visited the clinic, 230 (212 males, 18 females) underwent MRA (Table 1). With regard to the prevalence of underlying conditions in male patients, central nervous system disorders, including cerebrovascular diseases, were the most common (134/212, 63.2%), followed by gastric diseases (114/212, 53.8%) and esophageal diseases (106/212, 50.0%).

**TABLE 1:** Classification of causative conditions of hiccups^*^.

Clinical characteristics	Male		Female	
*n*	212		18	
Demographic data (mean ± SD)				
Age, years	61.2 ± 15.2		48.8 ± 20.7	
Height, cm	161 ± 67		155 ± 5	
Weight, kg	65.8 ± 9.3		51.7 ± 11.4	
Coexisting conditions, n (%)				
Central nervous system disorders	134	(63.2)	8	(44.4)
Cerebral infarctions Brainstem infarctions Cerebral hemorrhages Brain tumors Multiple sclerosis	46 20 15 5 3	(21.7) (9.4) (7.1) (2.4) (1.4)	1 0 0 1 0	(5.5) (0) (0) (5.6) (0)
White matter hyperintensities	48	(22.6)	2	(11.1)
Vagus or phrenic nerve irritation	7	(3.3)	0	(0)
Gastric etiologies	114	(53.8)	13	(72.2)
Esophageal etiologies	106	(50.0)	13	(72.2)
Intrathoracic etiologies	55	(25.9)	5	(27.8)
Postoperative etiologies	66	(31.1)	8	(44.4)
Other irritants	63	(29.7)	7	(38.9)
Medication‐induced	85	(40.1)	15	(83.3)
Toxic‐metabolic etiologies	2	(0.9)	0	(0)
Psychogenic etiologies	78	(36.8)	7	(38.9)
Lifestyle diseases, *n* (%)				
Hypertension	72	(34.0)	4	(22.2)
Hyperlipidemia	42	(19.8)	6	(33.3)
Diabetes	44	(20.8)	2	(11.1)

Abbreviation: SD, standard deviation.

^a^ Multiple conditions may exist in a single patient.

The types of cerebrovascular diseases in these patients were as follows: Cerebral infarction (46 cases), brainstem infarction (20 cases), deep white matter lesions (48 cases), and cerebral hemorrhage (15 cases). Among female patients, the most common cause of hiccups was a history of taking medications that could induce hiccups, such as benzodiazepine anti‐anxiety drugs (15/18, 83.3%). However, there were no cases in which these medications were thought to be the sole cause of hiccups. Gastric diseases (13/18, 72.2%) and esophageal diseases (13/18, 72.2%) were the next most common causes. Regarding cerebrovascular diseases, only one of the 18 female patients had a history of cerebrovascular diseases.

The distribution of CW types in the patient population was significantly different from that in each of the five healthy cohorts (Table 2). Of the 230 patients, 215 (93.5%) had an incomplete CW variant, whereas similar variants were consistently less frequent in healthy controls among the five cohorts from Japan (Ikeda et al. 2003), the Netherlands (Krabbe‐Hartkamp et al. 1998), Bosnia and Herzegovina (Voljevica et al. 2005), China (Li et al. 2011), and Taiwan (Chen et al. 2004) (*p* < 0.001 for all cohorts). Similarly, 195 (84.8%) of the 230 patients had a P‐com deficiency, which was significantly higher than that in any of the healthy cohorts.

**TABLE 2:** Comparison of the vascular distribution patterns of the Circle of Willis.

Analysis	Hiccup cohort	Control cohorts				
	Present study (*n* = 230)	Dutch (*n* = 150)	Bosnian (*n* = 150)	Taiwanese (*n* = 507)	Chinese (*n* = 160)	Japanese (*n* = 61)
All types	REF	*p* < 0.001	*p* < 0.001	*p* < 0.001	*p* < 0.001	*p* < 0.001
Type e vs. all non‐e types	140 (60.9% [54.2% to 67.2%]) vs. 90 (39.1% [32.8% to 45.8%]); REF	16 (10.7% [6.2% to 16.7%]) vs. 134 (89.3% [83.3% to 93.8%]); Δ = 50.2% (42.2% to 58.2%); *p* < 0.001	13 (8.7% [4.7% to 14.4%]) vs. 137 (91.3% [85.6% to 95.3%]); Δ = 52.2% (44.5% to 60.0%); *p* < 0.001	217 (42.8% [38.4% to 47.2%]) vs. 290 (57.2% [52.8% to 61.6%]); Δ = 18.1% (10.4% to 25.7%); *p* < 0.001	53 (33.1% [25.9% to 41.0%]) vs. 107 (66.9% [59.0% to 74.1%]); Δ = 27.7% (18.1% to 37.4%); *p* < 0.001	8 (13.1% [5.8% to 24.2%]) vs. 53 (86.9% [75.8% to 94.2%]); Δ = 47.8% (37.2% to 58.3%); *p* < 0.001
Complete type (types a–c) vs. incomplete type (types d–j)	15 (6.5% [3.7% to 10.5%]) vs. 215 (93.5% [89.5% to 96.3%]); REF	78 (52.0% [43.7% to 60.2%]) vs. 72 (48.0% [39.8% to 56.3%]); Δ = −45.5% (−54.1% to −36.9%); *p* < 0.001	81 (54.0% [45.7% to 62.2%]) vs. 69 (46.0% [37.8% to 54.3%]); Δ = −47.5% (−56.1% to −38.9%); *p* < 0.001	129 (25.4% [21.7% to 29.5%]) vs. 378 (74.6% [70.5% to 78.3%]); Δ = −18.9% (−23.9% to −14.0%); *p* < 0.001	46 (28.8% [21.9% to 36.4%]) vs. 114 (71.2% [63.6% to 78.1%]); Δ = −22.2% (−29.9% to −14.5%); *p* < 0.001	33 (54.1% [40.8% to 66.9%]) vs. 28 (45.9% [33.1% to 59.2%]); Δ = −47.6% (−60.5% to −34.7%); *p* < 0.001
P‐com deficiency (types d, e, g, and h) vs. non‐P‐com deficiency (types a–c, f, i, and j)	195 (84.8% [79.5% to 89.2%]) vs. 35 (15.2% [10.8% to 20.5%]); REF	67 (44.7% [36.6% to 53.0%]) vs. 83 (55.3% [47.0% to 63.4%]); Δ = 40.1% (30.9% to 49.3%); *p* < 0.001	63 (42.0% [34.0% to 50.3%]) vs. 87 (58.0% [49.7% to 66.0%]); Δ = 42.8% (33.6% to 51.9%); *p* < 0.001	351 (69.2% [65.0% to 73.2%]) vs. 156 (30.8% [26.8% to 35.0%]); Δ = 15.6% (9.4% to 21.7%); *p* < 0.001	88 (55.0% [46.9% to 62.9%]) vs. 72 (45.0% [37.1% to 53.1%]); Δ = 29.8% (20.8% to 38.8%); *p* < 0.001	27 (44.3% [31.5% to 57.6%]) vs. 34 (55.7% [42.4% to 68.5%]); Δ = 40.5% (27.2% to 53.8%); *p* < 0.001
Fetal type (types b, c, and f–j) vs. non‐fetal type (types a, d, and e)	66 (28.7% [22.9% to 35.0%]) vs. 164 (71.3% [65.0% to 77.1%]); REF	48 (32.0% [24.6% to 40.1%]) vs. 102 (68.0% [59.9% to 75.4%]); Δ = −3.3% (−12.8% to 6.2%); *p* = 0.49	52 (34.7% [27.1% to 42.9%]) vs. 98 (65.3% [57.1% to 72.9%]); Δ = −6.0% (−15.6% to 3.6%); *p* = 0.22	191 (37.7% [33.4% to 42.1%]) vs. 316 (62.3% [57.9% to 66.6%]); Δ = −9.0% (−16.2% to −1.8%); *p* = 0.018	117 (73.1% [65.6% to 79.8%]) vs. 43 (26.9% [20.2% to 34.4%]); Δ = −44.4% (−53.4% to −35.4%); *p* < 0.001	12 (19.7% [10.6% to 31.8%]) vs. 49 (80.3% [68.2% to 89.4%]); Δ = 9.0% (−2.5% to 20.6%); *p* = 0.16

*Note*: Data are shown as the numbers (percentage [95% CI]) for those with versus without a target variant pattern followed by the difference Δ (95% CI) in the percentage of the target variant pattern between the hiccup (reference) and control cohorts; and *p* values.

Abbreviations: CI, confidence interval; P‐com, posterior communicating artery; REF, reference.

The frequency of the fetal types was significantly lower in our cohort than in the cohorts from China and Taiwan (66/230 [28.7%] in our hiccup cohort vs. 117/160 [73.1%] and 191/507 [37.7%] in the China and Taiwan cohorts; *p* < 0.001 and *p* = 0.018, respectively). Among all variants, type e—characterized by bilateral P‐com hypoplasia or absence, resulting in anterior‐posterior CW isolation—was the most common (140/230, 60.9%). The frequency of the type e variant in our patients was significantly higher than that in each of the healthy cohorts (*p* < 0.001 for all cohorts).

The small number of female patients precluded a similar subgroup analysis. P‐com deficiency variants were found in 58 (95.1%) of 61 patients with cerebrovascular events, including 20 patients with brainstem infarctions, which was comparable to that of the entire cohort.

## 4 Discussion

In this single‐center retrospective case series study of intractable hiccups in Japanese patients, more than 90% of patients had incomplete CW (45%–75% in the healthy cohort), and 85% had P‐com deficiency (30%–55% in the healthy cohorts).

Type‐e bilateral P‐com deficiency was the most common, seen in 60% of patients, compared with 10%–40% of individuals in healthy cohorts.

As shown in Figure 1, the CW is defined as complete when it forms a complete ring, and incomplete when the ring is partially missing. There has been much discussion about the role of this ring, and it was previously thought to play a role in compensating for blood flow (circulatory security) when some type of ischemic change occurs in a certain area (Symonds 1955), but recently it has been thought that the CW plays a role in protecting cerebrovascular disease as a scavenger when a sudden change in perfusion pressure (whether increasing or decreasing) occurs (Bloomfield et al. 1997). Although the incidence of complete CW varies among studies, it is generally considered approximately 36%–55% (Hartkamp et al. 1999; Macchi et al. 2002).

Although there could be racial differences in the distribution of CW variants, the frequency of type e variants in the patients in this study was higher than in all other healthy cohorts that were studied, including East Asian cohorts planned with racial differences in mind. Patients with type e CW vascular variants may be prone to intractable hiccups. The frequency of the fetal type was significantly lower in our cohort than in the Chinese and Taiwanese cohorts, suggesting that fetal vascular variants are less associated with intractable hiccups.

A hiccup is a reflex movement with stimulatory receptors in the dorsal nasopharynx, and the pharyngeal branches of the glossopharyngeal nerve has been identified as the afferent pathway (Kondo et al. 2003), a center in the reticular formation near the nucleus ambiguus in the medulla oblongata (Arita et al. 1994). It is known that GABA plays an important role in controlling the reflex (Oshima et al. 1998). However, the higher brain areas that control the hiccup center are unknown. Recently, it has been reported that the insular cortex, temporal lobe, and subcortical area are suggested as the higher centers of the hiccup reflex (Itabashi et al. 2019), and that there are projection pathways from the thalamus to the insular cortex (Ramsay et al. 2023). These findings suggest that the insular cortex, temporal lobe, subcortical areas, and thalamus probably control the hiccup center in the medulla oblongata.

Arteries branching from the P‐com perfuse the third ventricle, pituitary trunk, optic chiasm, thalamus, hypothalamus, and internal capsule, but not the medulla oblongata. Therefore, if hiccups occur due to changes in blood flow related by P‐com deficiency, it seems reasonable to assume that the changes in blood flow do not act directly on the medulla oblongata, but rather occur through effects on the areas controlling the medulla oblongata. To clarify this mechanism, direct evaluation of blood flow and brain activity using f‐MRI in areas perfused by P‐com, such as the thalamus and insular cortex, seems to be necessary.

Future studies are necessary to validate the observed differences in CW variant type patterns on MRA images, which account for important underlying risk factors, including cerebral infarction. In cases of intractable hiccups developing after cerebral infarction in a specific part of the brain (such as the thalamus), comparing MRI/MRA images before and after the onset of hiccups would provide valuable information.

This study is a single‐center, retrospective, observational study using 1.5 Tesla phase‐control MRA, which is a limitation of this study. The morphology of the P‐com observed in this study is congenital and may differ from acquired occlusions or stenoses. Given the high frequency of cerebrovascular disease in our hiccup cohort, a cohort with a history of cerebral infarction but without intractable hiccups would be a good candidate for control group.

## Author Contributions

**Tsukasa Kondo**: conceptualization, data curation, formal analysis, investigation, methodology, project administration, resources, supervision, validation, visualization, writing – original draft, writing – review and editing. **Teruhiko Terasawa**: conceptualization, formal analysis, investigation, methodology, software, validation, writing – review and editing. **Hideyuki Nakama**: conceptualization, investigation, validation, writing – review and editing.

## Ethics Statement

This study was approved by the Ethics Committee of the Yuai Memorial Hospital and conformed to the guidelines set in the Declaration of Helsinki. (Approved No. 20180703)

## Consent

All participants provided informed written consent prior to participation in the study.

## Conflicts of Interest

The authors declare no conflicts of interest.

## Peer Review

The peer review history for this article is available at https://publons.com/publon/10.1002/brb3.70978.

## Data Availability

The datasets used and/or analyzed during the current study are available from the corresponding author on reasonable request.
